# Seventh Year

**Published:** 1871-04

**Authors:** 


### The Bistoury.

ELMIRA, N. Y., APRIL, 1871.

The BISTOURY is published Quarterly, upon the 1st of April, July, October and January, at Fifty Cents a year, in advance.

### SEVENTH YEAR.

THE BISTOURY has now entered upon the seventh year of its existence, and is a visitor in every State and Territory. Its success has been much greater than we hoped for. We have received very many letters, from all parts of the country, assuring us that our work is appreciated, and urging us to continue in the course we have taken. Many ladies and gentlemen have interested themselves in our behalf by sending us large lists of subscribers, thus giving us practical assistance and support. To all such we return thanks, and will strive to merit their esteem in the future, as we have endeavored to in the past. We club with all the principal Magazines in the country, offering THE BISTOURY free to all who will order their journals through us. We also offer large cash premiums to those who will canvass for our magazine. The list will be sent to all who apply for it. Those subscribers who are in arrears for subscription will be again reminded of the fact, by the envelope enclosed in this number. We hope they will fill out the blank and enclose the amount due us—the sum is small, therefore the easier paid. Read the first number of volume seven carefully, and see whether we have not given you information worth many times more than is asked for it.

